# Anticancer drugs approved by the Food and Drug Administration for gastrointestinal malignancies: Clinical benefit and price considerations

**DOI:** 10.1002/cam4.2058

**Published:** 2019-03-07

**Authors:** Di Maria Jiang, Kelvin K. W. Chan, Raymond W. Jang, Christopher Booth, Geoffrey Liu, Eitan Amir, Robert Mason, Louis Everest, Elena Elimova

**Affiliations:** ^1^ Division of Medical Oncology, Princess Margaret Cancer Centre University Health Network, University of Toronto Toronto Canada; ^2^ Division of Medical Oncology & Hematology, Odette Cancer Centre, Sunnybrook Health Sciences Centre University of Toronto Toronto Canada; ^3^ Canadian Centre for Applied Research in Cancer Control Toronto Canada; ^4^ Dalla Lana School of Public Health University of Toronto Toronto Canada; ^5^ Department of Oncology Queen’s University Kingston Ontario Canada; ^6^ Division of Cancer Care and Epidemiology Queen’s Cancer Research Institute Kingston Ontario Canada

**Keywords:** antineoplastic agents, clinical benefit, drug costs, gastrointestinal neoplasms, United States Food and Drug Administration

## Abstract

**Background:**

The cost of new anticancer drugs is rising. We aimed to assess the clinical benefit and price of anti‐cancer drugs approved by the US Food and Drug Administration (FDA) for advanced gastrointestinal cancers.

**Methods:**

Drugs approved between 2006 and 2017 for advanced GI malignancies were identified from FDA.gov, and their updated supporting trial data were searched. Incremental clinical benefit was quantified by using ESMO Magnitude of Clinical Benefit Scale version 1.1 (grade 0‐5) and ASCO Value Framework version 2 (score range −20 to 180). Higher scores indicate larger net benefit, and substantial benefit was defined as score 4 or 5 by the European Society for Medical Oncology (ESMO). The Micromedex REDBOOK was used to estimate the monthly average wholesale price (AWP) and total drug price (TDP) over the median treatment duration per patient. Clinical benefit, AWP and TDP of each drug class were assessed.

**Results:**

In total, 16 GI cancer drugs received FDA approval for 24 indications, including five monoclonal antibodies (mAbs), five oral targeted therapies (TT), two immunotherapeutics (IO), three cytotoxic chemotherapies (CT), and one recombinant fusion protein (aflibercept). Most supporting trials (82%) reported overall survival benefit of less than 3 months and no significant improvement in quality of life. Only five agents (including one TT and one IO) with 21% the of approved indications met the ESMO's threshold of substantial clinical benefit. Median incremental benefit scores of TT and IO were comparable to other drug classes. However their median TDP was much higher at $153 402 and $98 208, respectively, compared to $30 330 USD per patient for CT. The estimated TDP did not correlate with clinical benefit scores.

**Conclusion:**

Most FDA–approved gastrointestinal cancer drugs do not meet the ESMO threshold of substantial clinical benefit. TT and IO are estimated to carry significant drug costs, and further cost analysis of these drugs is urgently needed.

## 1 INTRODUCTION

By 2020, the cost of cancer care in the United States is expected to reach $158 billion US dollars.^1^ Pharmaceutical drugs are the fastest growing aspect of US health care spending, with oncology treatments amounting to nearly $37.8 billion in 2015.^2^, ^3^ Over the past decade, the average monthly price of oncology drugs more than doubled, from $7 103 to $15 535 USD.^4^ Concerns arise as the unprecedented increase in price of new anticancer drugs seem disproportionate to the clinical benefit they produce.^5^, ^6^

To address the growing disconnect between clinical benefit and cost, various frameworks have been developed by organizations including the American Society of Clinical Oncology (ASCO) and the European Society for Medical Oncology (ESMO).^9^, ^10^ The ASCO value framework (VF) was designed to standardize the assessment of value for new anticancer treatment to guide decision‐making by physicians and patients.^9^, ^14^ ESMO intends to use the Magnitude of Clinical Benefit Scale (MCBS) to select anti‐cancer therapies that offer the greatest benefit, and prioritize their drug funding across the European Union.^10^ Notably, more than half of the accelerated US Food and Drug Administration (FDA) approval indications of new anticancer drugs were based on single–arm studies and could not be assessed with the original ESMO MCBS 15, 16; however these can now be evaluated with ESMO MCBS version 1.1.^17^

GI oncology has seen a tremendous pace of approvals for novel therapeutic agents including targeted therapies (TT) and immunotherapeutics (IO). In metastatic colorectal cancer (CRC), median survival has nearly doubled over the last decade. However, this has been accompanied by a staggering 340‐fold increase in drug costs.^6^ CRC has now become the second most expensive cancer to treat.^1^ Recent work has shown only modest activity of immunotherapy in GI tumors except in tumors with high microsatellite instability.^18^ The majority of GI tumors are less immunogenic than malignancies such as lung cancer and melanoma. Unlike TT in lung cancer in which distinct driver pathways and predictive biomarkers such as EGFR have been identified, treatment of GI cancers often rely on multityrosine kinase inhibitors and monoclonal antibodies without the ability to predict responders. In light of these findings and the current emphasis on value in cancer care, we aimed to quantify the net clinical benefit and price of newly FDA–approved anticancer drugs for advanced GI malignancies using ASCO VF and ESMO MCBS, with a special focus on TT and IO.

## 2 MATERIALS AND METHODS

### 2.1 Data identification

**Figure 1:**
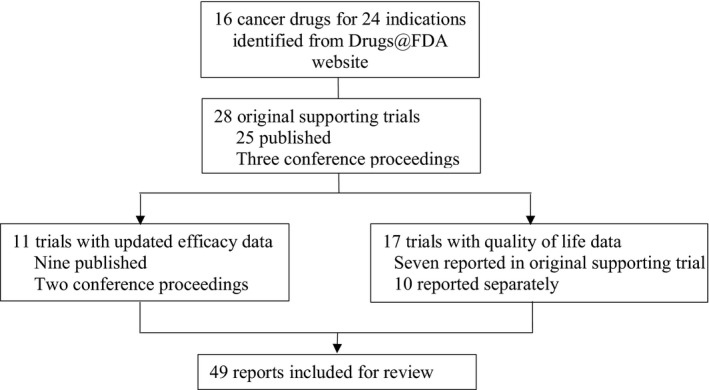
Identification of trials for US Food and Drug Administration (FDA)–approved new anticancer drugs for treatment of advanced or metastatic GI cancers

The Drugs@FDA website^19^ was accessed on 27 January 2018 to identify new anticancer drugs approved between 1 January 2006 and 31 December 2017 for treatment of advanced or metastatic GI cancers. We excluded anticancer drugs approved for pediatric populations and supportive care drugs (eg, antiemetics and growth factors). Approved anti‐GI cancer drugs were classified into five groups: monoclonal antibodies (Mab, eg, cetuximab); oral targeted therapies (TT, eg, sunitinib); immunotherapeutics (IO, eg, nivolumab); cytotoxic chemotherapies (CT, eg, nab‐paclitaxel); and other (aflibercept). Their supporting trials were identified through PubMed, ASCO and ESMO conference proceedings (Figure 1). Updated data on efficacy endpoints and companion quality of life (QOL) publications were searched and included whenever available.

### 2.2 Quantifying incremental clinical benefit

Incremental clinical benefit was quantified using ESMO MCBS version1.1 2017 (grade 0‐5) and ASCO VF version2 2018 (range −20 to 180), with higher scores indicating larger net benefit.^14^, ^17^ Only the most recently published updated trial data are used for scoring. For trials evaluating EGFR inhibitors, only outcomes of wild‐type KRAS tumors were assessed. ESMO uses the lower limit of the 95% confidence interval (CI) of the HR and the absolute difference in outcomes to determine clinical benefit, with toxicity or QOL parameters used as modifiers. Substantial benefit was defined as score 4 or 5 by ESMO MCBS;^10^ no specific definition was outlined by the ASCO VF. The net clinical benefit score in ASCO VF is derived from hazard ratios and absolute values of OS, PFS, and response rates in the order as previously described.^14^ Toxicity scores were determined using the most recently published trial data, including table and text descriptions of adverse events. Bonus points are awarded for tail‐of‐the‐curve effect (suggesting possible long–term survival), treatment–free interval, and palliation of symptoms or QOL benefit. Unlike ESMO MCBS, single–arm trials without comparator arms are not meant to be assessed using ASCO VF.^9^, ^14^

One author (DMJ) extracted and scored all available data from the included studies. Subsequently, two authors (RM, LE) assessed the extracted data independently (each responsible for half of the studies included). A fourth author (KKWC) resolved any disagreement.

### 2.3 Estimation of drug cost

ASCO VF also incorporates drug acquisition cost (DAC) and patient out‐of‐pocket costs including supportive medications, which are highly dependent on insurance coverage thus difficult to measure and were not included in our study. DAC is based on the average sale price for intravenous medications and information from United Healthcare for oral medications.^14^ Other costs such as those related to hospitalization and loss of productivity are excluded.

The Micromedex REDBOOK (accessed in February 2018) was used to determine monthly average wholesale pricing (AWP) and estimate the total drug price (TDP) for each drug. The REDBOOK is an online database of unbiased AWP which publishes manufacturer–reported drug price.^20^ Monthly AWP was calculated according to FDA–approved dosing, assuming an average patient weighing 70 kg with a body surface area (BSA) of 1.73 m^2^.^21^ Regimens not delivered as monthly cycles were adjusted to provide the average dose used per 4‐week period. We did not calculate incremental monthly drug price. TDP was estimated by multiplying the monthly AWP by the reported median duration of treatment. Median progression–free survival (PFS) was substituted if the former is not reported (or PFS at the lower CI if median PFS was not reached).

### 2.4 Data analysis

Data were collected in Microsoft Office Excel 2010. Data were reported descriptively as proportions, medians, and ranges where appropriate. Scatterplots were used to show the relationship between benefit scores and cost, and spearman correlation was used to quantify their correlation, using online SAS® Studio 3.71 (Cary, NC, USA). Interrater reliability was assessed using the intraclass correlation coefficient (ICC), with a value of 0 demonstrating no agreement and a value of 1 demonstrating perfect agreement. All statistical tests were two‐sided, and statistical significance was defined as a two‐sided *P* value of less than 0.05.

## 3 RESULTS

### 3.1 FDA approvals in advanced or metastatic GI cancers

Between 2006 and 2017, 16 new drugs received FDA approval for treatment of advanced GI cancers. There were 24 indications based on 28 supporting trials. Among these, there were five Mab's (cetuximab, panitumumab, bevacizumab, ramucirumab, and trastuzumab), five oral TTs (sorafenib, regorafenib, lanreotide, sunitinib and everolimus), two IO's (nivolumab and pembrolizumab), three CTs (TAS‐102, nab‐paclitaxel, and liposomal irinotecan), and one recombinant fusion protein (aflibercept). CT only represented 11% (3/24) of all approvals. TT and IO trials represented 50% of the 28 supporting trials (25% for TT, 25% for IO) (see Tables 1 and 2).

**Table 1:** New anticancer drugs approved by the FDA between 2006 and 2017 for treatment of advanced GI cancers and their most recent supporting trials

Trial characteristics (n = 28)	n (%)
Disease site	
CRC	10 (36)
MSI‐H or deficient MMR	5 (18)
Gastroesophageal	4 (14)
NET	4 (14)
HCC	3 (11)
PDAC	2 (7)
Classes of therapy	
Mab	10 (36)
TT	7 (25)
IO	7 (25)
CT	3 (11)
Other (aflibercept)	1 (4)
Approved line of therapy	
1	11 (40)
2	12 (43)
≥3	5 (18)
Type of trial	
Phase I	2 (7)
Phase II single arm	5 (18)
Phase II RCT	1 (4)
Phase III RCT	20 (71)
Primary endpoint	
OS	14 (50)
QOL	0
PFS	7 (25)
ORR	8 (29)
Benefit in OS	15 (54)
Benefit in QOL	4 (14)

CRC, colorectal cancer; CT, cytotoxic chemotherapy; HCC, hepatocellulcar carcinoma; IO, immunotherapy; Mab, monoclonal antibody; MMR, mismatch repair; MSI‐H, microsatellite instability–high cancer; NET, neuroendocrine tumors; ORR, objective response rate; OS, overall survival; PDAC, pancreatic ductal adenocarcinomas; PFS, progression–free survival; QOL, quality of life; RCT, randomized control trial; TT, oral targeted therapies.

**Table 2:** New anticancer drugs approved by the FDA for treatment of advanced or metastatic GI cancers between 2006 and 2017, their most recent supporting trial data, incremental clinical benefit scores and estimated drug cost

Cancer	Trial year	Phase	First (1) or subsequent (2) approval	Line of therapy	n	Treatment	FDA approval year	Primary endpoint	HR	95% confidence interval	Absolute gain (months, or % for ORR)	OS benefit	QOL benefit	ASCO VF NHB version2	ESMO MCBS CBS version1.1	Monthly Drug AWP (USD)	Median treatment duration (months)	Estimated drug cost (USD) per patient
**CRC**	OPUS 2011	II	2	1	179	Cetuximab ± FOLFOX4	2012	ORR	2.55^a^	1.38‐4.72	23%	None	None	9.2	1	$12 591.37	8.30	$102 052.55
	CRYSTAL 2015	III	2	1	430	Cetuximab ± FOLFIRI		PFS	0.56	0.41‐0.76	8.2	Yes^b^	None	11.0	4	$12 591.37	11.40	$141 085.80
	**CO.17** **2008**	**III**	**2**	**≥3**	394	**Cetuximab vs** **BSC**	**2007**	**OS**	**0.55**	**0.41‐0.74**	**4.7**	**Yes**	**Yes**	**71.8**	**5**	**$12 591.37**	**3.70**	**$44 132.24**
	Amado 2008	III	1	≥3	427	Panitumumab vs BSC	2006	PFS	0.45	0.34‐0.59	1.3	None	None	43.0	1	$11 969.37	3.08	$36 805.82
	ECOG E3200 2007	III	2	2	829	Bevacizumab ± FOLFOX4	2006	OS	0.75	NA	2.1	Yes	None	19.7	1	$13 062.00	5.00	$65 310.00
	ML18147 2013	III	2	2	409	Bevacizumab ± chemotherapy	2013	OS	0.81	0.69‐0.94	1.4	Yes	None	18.2	1	$6 531.00	3.90	$25 470.90
	RAISE 2016	III	2	2	1072	Ramucirumab ± FOLFIRI	2015	OS	0.84	0.73‐0.98	1.6	Yes	None	29.6	1	$14 914.37	4.75	$70 843.25
	VELOUR 2012	III	1	2	1226	Aflibercept ± FOLFIRI	2012	OS	0.82	0.71‐0.94	1.4	Yes	None	16.0	1	$10 752	3.50	$37 632.00
	CORRECT 2013	III	1	≥3	760	Regorafenib vs placebo	2012	OS	0.77	0.64‐0.94	1.4	Yes	None	4.4	1	$15 625.56	2.80	$43 751.57
	RECOURSE 2015	III	1	≥3	800	TAS‐102 vs placebo	2015	OS	0.68	0.58‐0.81	1.8	Yes	None	49.4	2	$15 164.98	2.00	$30 329.96
Gastric	RAINBOW 2014	III	2	2	665	Ramucirumab ± paclitaxel	2014	OS	0.81	0.68‐0.96	2.2	Yes	Yes	38.7	2	$14 914.37	4.50	$67 114.66
	REGARD 2014	III	1	≥2	355	Ramucirumab vs placebo	2014	OS	0.78	0.60‐0.998	1.4	Yes	None	36.5	1	$14 914.37	2.10	$31 320.17
**HER2+ Gastric**	**TOGA 2010**	**III**	**2**	**1**	594	**Trastuzumab** ± **CF/CX**	**2010**	**OS**	**0.74**	**0.60‐0.91**	**2.7**	**Yes**	**Yes**	**33.1**	**4**	$6 778.37	4.90	$28 690.22
PDL1+ gastric	KEYNOTE 059 2017	II	2	≥3	259	Pembrolizumab	2017	ORR	—	11%‐23%	16%	None	None	—h	1	$14 658.93	14.20	$216 952.21
**MSI‐H CRC**	**CHECKMATE 142 2017**	**II**	**2**	**≥2**	74	**Nivolumab**	**2017**	**ORR**	—	**23%‐46%**	**34%**	**None**	**Yes**	—h	**4**	$14 879.98	6.60	$98 207.87
MSI‐H Ca	Le 2017	II	2	≥2	86	Pembrolizumab	2017	ORR PFS	— — —	36%‐68%^c^ 39‐69^d^ —	52%^c^ 54%^d^ NR	None	None	—h	3	$14 658.93	>14.80^f^	$>216 952.21^e^
	KEYNOTE 158 164 2017	II	2	≥2	138	Pembrolizumab		ORR	—	17%‐41%^c^ 27%‐49%^d^	37.7%^c^ 27.9%^d^	None	None	—h	2	$14 658.93	>6.57^g^	$>98 947.80^e^
	KEYNOTE 012 2017	Ib	2	≥2	39	Pembrolizumab		ORR	—	10%‐39%	22%	None	None	—h	1	$14 658.93	2.00	$29 317.87^d^
	KEYNOTE 028 2017	Ib	2	≥1	33	Pembrolizumab		ORR	—	—	4%	None	None	—h	0	$14 658.93	1.80	$26 386.08
**HCC**	SHARP 2008	III	2	1	602	Sorafenib vs placebo	2007	OS, TTP^c^	0.69	0.55‐0.87	2.8	Yes	None	45.6	3	**$20 764.08**	**5.30**	**$110 049.62**
	**RESORCE** **2017**	**III**	**2**	**2**	573	**Regorafenib vs** **placebo**	**2017**	**OS**	**0.63**	**0.50‐0.79**	**2.8**	**Yes**	**None**	**37.0**	**4**	$15 625.56	3.60	$56 252.02
	CHECKMATE 040 2017	I/II	2	≥1	262	Nivolumab	2017	ORR	—	—	20%	None	None	—h	3	$14 879.98	4.00	$59 519.92
pNET	CLARINET 2014	III	2	1	204	Lanreotide vs placebo	2014	PFS	0.47	0.30‐0.73	NR	None	None	22.4	3	$8 698.80	40.00	$347 952.00
	SUN1111 2016	III	2	≥1	160	Sunitinib vs placebo	2011	PFS	0.32	0.18‐0.55	6.8	None	None	38.0	3	$17 597.39	11.40	$200 610.25
	RADIANT‐3 2016	III	2	≥1	410	Everolimus vs placebo	2011	PFS	0.35	0.27‐0.45	7.4	None	None	2.0	2	$17 451.90	8.79	$153 402.20
pNET or lgNET	RADIANT‐4 2016	III	2	≥1	302	Everolimus vs placebo	2016	PFS	0.48	0.36‐0.67	7.1	None	None	32.0	2	$17 451.90	10.10	$176 264.19
PDAC	Von Hoff 2013	III	2	1	861	Nab‐paclitaxel ± gemcitabine	2013	OS	0.72	0.62‐0.83	1.8	Yes	None	41.6	2	$9 787.56	3.90	$38 171.49
	NAPOLI‐1 2016	III	1	2	417	Liposomal irinotecan ± 5FU	2015	OS	0.67	0.49‐0.92	1.9	Yes	None	49.8	2	$12 037.90	2.18	$26 182.44

Bold: meet the ESMO threshold of meaningful clinical benefit.

ASCO VF, ASCO value framework version 2 2016 14; AWP, average wholesale price; BSC, best supportive care; CBS, clinical benefit scale; CRC, colorectal cancer; ESMO MCBS, ESMO magnitude of clinical benefit version 1.1 2017 17; FDA, US Food and Drug Administration; HCC, hepatocellulcar carcinoma, lgNET, lung neuroendocrine tumors; MSI‐H, microsatellite instability–high cancer; NA, not available; NHB, net health benefit; ORR, objective response rate; OS, overall survival; PDAC, pancreatic ductal adenocarcinomas; PFS, progression–free survival; pNET, pancreatic neuroendocrine tumors; QOL, quality of life; TTP, time to symptomatic progression.

a Odds ratio.

b OS HR 0.69 (95% CI 0.54‐0.88), absolute benefit 8.2 months.

c In MSI‐H nonCRC.

d In MSI‐H CRC.

e Median treatment duration or PFS not reached.

f Median PFS not reached (95% CI 14.8 months to not reached) in the updated analysis published in 2017.

g Median follow up 27 weeks for keynote 158 (54 weeks for keynote 164). Median duration of treatment not reported, median PFS not reported, median duration of response was not reached.

h ASCO VF not presented as it is not meant to evaluate single–arm studies without comparator arms.

The 28 supporting trials included 20 (71%) phase III randomized controlled trials (RCTs) with a median sample size of 502 patients. OS was the primary endpoint in 14 studies (50%). No trial used QOL as the primary endpoint. Surrogate primary endpoints (ORR, PFS) were used by 54% of the studies, with two studies used coprimary endpoints (SHARP trial 2008 and Le 2017). There were seven single–arm studies (25%) which led to accelerated approval of two IO agents for four indications.

### 3.2 Incremental clinical benefit and price considerations

Overall, 54% of studies demonstrated a statistically significant improvement in OS (see Table 2, which displays individual clinical benefit scoring components). The median improvement in OS was 1.9 months (range 1.3‐4.7 months) and HR OS of 0.74 (range 0.55‐0.84). Out of the 14 trials conducted using surrogate primary endpoints, only one demonstrated superior OS (CRYSTAL 2015), and none demonstrated QOL improvements. QOL outcomes were reported in 17 trials (61%); only four (24%) of which demonstrated significant benefit (Table 2). A vast majority of trials (82%) did not achieve an OS benefit of 3 months or demonstrate any improvement in QOL.

Among the 24 approved indications, five (21%) met the ESMO criteria for substantial benefit (grade 4‐5): Cetuximab (CO.17 trial, CRYSTAL trial), Trastuzumab (TOGA trial), Nivolumab (Checkmate 142 trial), and Regorafenib (RESORCE trial). All showed either OS or QOL benefit. Cetuximab in pretreated KRAS wild‐type CRC patients achieved the highest incremental clinical benefit (ESMO MCBS 5, ASCO VF 71.8) by achieving a relatively large OS (4.7 months, HR 0.55) and significant QOL benefit (see Table 2, which displays individual clinical benefit scores and drug prices).

The two scales demonstrated a moderate correlation with their net benefit scores (spearman correlation coefficient 0.47, *P* = 0.012). Interrater reliability was high. ICC values were 0.96 (95% CI 0.91‐0.98, *P* < 0.001) and 0.94 (95% CI 0.79‐0.96, *P* < 0.001) for ASCO NHB and ESMO MCBS scores respectively, and Kappa scores of 0.73 for ESMO MCBS scores. Both scales suggest the incremental clinical benefit of anti‐GI cancer drugs have remained stable over the last decade (see Figure S1, which displays benefit scores over time).

**Figure 2:**
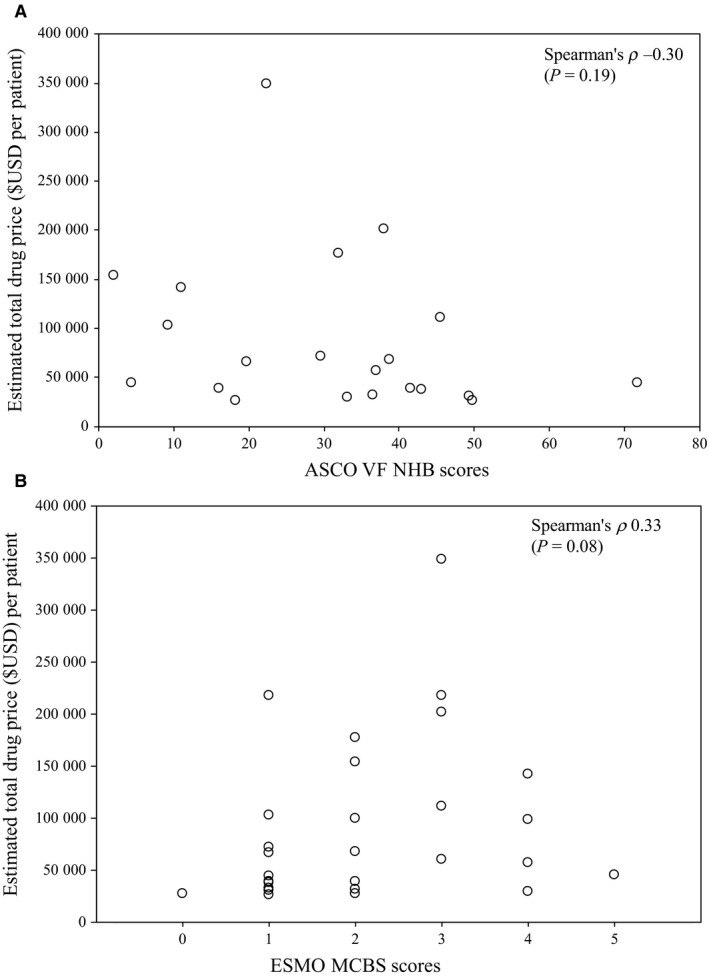
Scatterplot of correlation between ASCO VF net health benefit scores (A), ESMO MCBS clinical benefit scores (B) and estimated drug cost of FDA–approved GI oncology drugs per patient

Median monthly AWP was $14 769 USD per patient, and ranges from $6 531 (bevacizumab) to $20 764 (sorafenib). Estimated TDP ranges between $25 470 (bevacizumab) and $347 952 USD per patient (lanreotide). Median TDP was $62 415, with ten approvals (42%) exceeding $100 000 USD per patient (see Table 2, which displays individual clinical benefit scores and drug prices). Estimated TDP (Figure 2) and monthly AWP (data not shown) did not correlate with net clinical benefit scores.

A majority of approved anticancer drugs already have existing approvals in other disease sites (subsequent approval indications), while six indications represent first approval indications. The median ASCO VF NHB, ESMO MCBS score and estimated TDP for anticancer drugs with first vs subsequent approved indications were 31.8 vs 21.1, 1 vs 2, and $34 063 vs $84 526 respectively.

### 3.3 TT and immunotherapy agents

**Figure 3:**
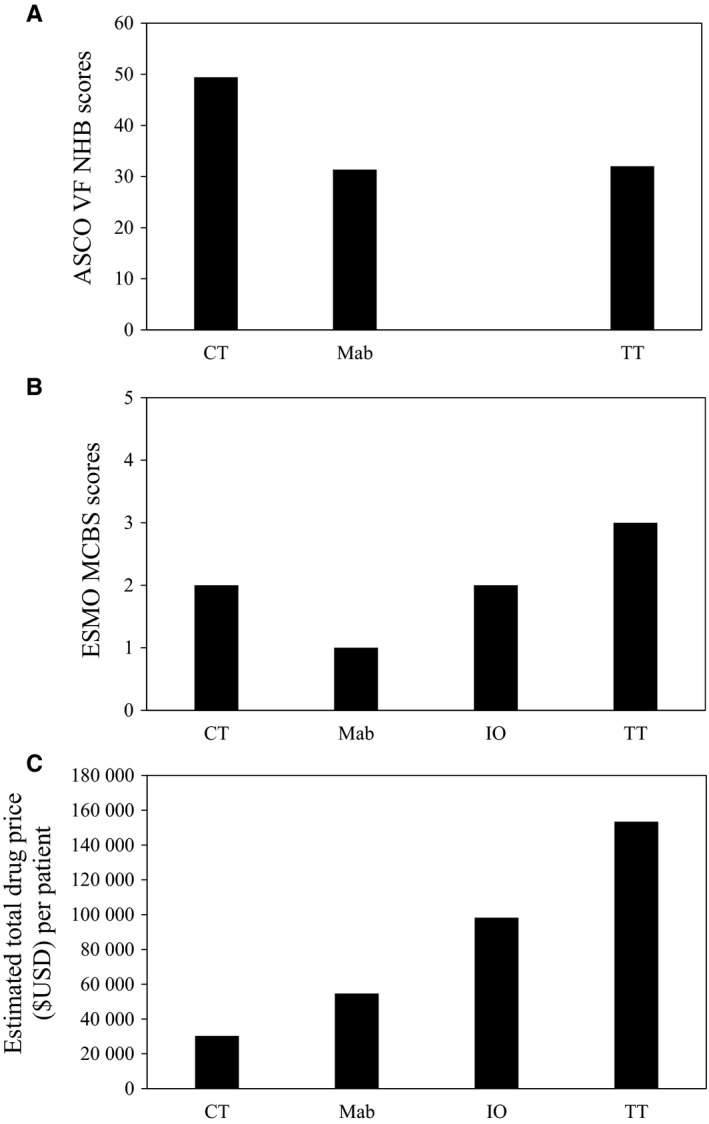
Median ASCO NHB (A), ESMO MCBS scores (B) and total drug cost per patient (C) of FDA–approved cytotoxic chemotherapies (CT), monoclonal antibodies (Mab), immunotherapeutics (IO) and targeted therapies (TT) for advanced GI cancers

Among the four classes of anticancer drugs, the median incremental benefit scores were comparable but their estimated TDP were highly variable (Figure 3). Median TDP of TT was the highest at $153 402 USD per patient and five times of that of CT, due to both higher monthly AWP and longer median treatment duration (see Table S1, which compares monthly AWP, treatment duration and TDP). Prolonged treatment durations over 10 months were seen in two IO, three TT, and one Mab trials (see Table 2, which displays treatment durations). Due to small sample sizes, statistical tests could not be applied to detect any differences of median benefit scores and estimated TDP between drug classes.

## 4 DISCUSSION

The present study identifies only a modest incremental benefit in most FDA–approved anti‐GI cancer drugs over preexisting therapies, including TT and IO. Although these drugs are proven to have significant and meaningful benefit for many patients, the magnitude of improvement as a whole is mostly marginal. Most supporting trials (82%) reported OS benefit of less than 3 months and no significant QOL improvement. Our study focused specifically on the GI disease site and found that even with inclusion of most recently published IO studies, most approved drugs enter the market with marginal incremental benefit and high drug prices. The magnitude of incremental clinical benefit of newly–approved cancer drugs over preexisting therapies is out of proportion with their extremely high drug prices. Comparing drug classes, TTs had the highest drug prices, surpassing that of IO.

We used the ASCO VF and ESMO MCBS to assess relative clinical benefit for which these drugs received FDA approval. Few anticancer drugs met the substantial benefit defined by ESMO. To achieve this threshold, generally speaking, a trial is required to demonstrate either a HR less than 0.65 at lower bound of 95% CI, or 3‐month gain in OS. Our results echo previous findings. Only 38.8% of FDA‐approved palliative anticancer drugs,^15^ and 35% of TT and biologics meet the ESMO substantial benefit threshold.^16^ Similarly, only 11% of the anticancer drugs approved by the European Medicines Agency meet this threshold,^22^ and 52% of the drugs do not improve OS or QOL.^23^ It is important to highlight that ASCO VF NHB scores should not be interpreted as an absolute measure of value, since it measures the incremental benefit of the experimental relative to the comparator arm. Although ASCO VF does not specify a threshold, Ellis et al recommended targets, including a 3‐5 month OS improvement for metastatic CRC.^24^ Only two out of nine approved indications in CRC (22%) met this modest target in our study. More recently, ASCO has proposed pragmatic threshold scores of 40 or less for low benefit, and 45 or greater for substantial benefit.^25^ Only four supporting trials met this threshold.

TT has previously demonstrated seemingly large magnitude of clinical benefit relative to their monthly market prices compared to chemotherapy.^5^, ^26^ While TT has significantly advanced treatment outcomes for many malignancies many of which are resistant to CT, the magnitude of incremental benefit of TT over preexisting therapies is similarly marginal compared to other drug classes. Most TTs demonstrated improvement in PFS only. Only regorafenib for HCC (one out of seven TTs) met the ESMO threshold of substantial benefit, and only one out of seven TT's (sorafenib for HCC) met the pragmatic threshold of substantial benefit proposed by ASCO. Yet TTs had significantly higher monthly AWP and TDP than other drug classes. Similarly, IO agents rarely meet ESMO's thresholds of substantial benefit (ASCO VF not applied to supporting trials of IO as it is not meant to evaluate single–arm studies), yet seem to incur significant drug prices. Further in depth cost analysis are urgently needed for TTs and IO.

We present exceedingly conservative estimates of drug prices of these newly–approved anti‐GI cancer drugs. The weight (70 kg) and BSA (1.73 m^2^) of an average patient assumed may be considerable underestimates. The average BSA in the contemporary patient population may be up to 1.86 m^2^.^27^ For cetuximab, using this higher BSA value will increase monthly AWP by $946 USD per patient. Additionally, many IO trials to date have not reached median duration of therapy used to estimate TDP. Substantial drug costs can significantly affect patients' treatment adherence and outcomes.^28^, ^29^ From a societal perspective, growing expenditures on anticancer drugs can potentially affect funding of other life–saving therapies. It is important to highlight that the drug prices we have presented are not the true costs. AWP does not represent the true drug cost, given the discounted bulk purchase pricing often offered by manufacturers. These important cost data unfortunately are mostly unavailable. Price negotiations also vary between institutions and may be modified over time with updated efficacy data and new indications.

The ASCO VF and ESMO MCBS represent important steps in the conversation regarding value of cancer therapies. However, they are imperfect tools and have been criticized for not addressing factors that affect efficacy outcomes (crossover, subsequent therapies, inadequate follow up), underreporting of adverse events in trials, and inability to assess the methodological strength of evidence.^30^, ^31^ Durable survival and response rates of modern IO therapy are also not fully recognized by these frameworks.^32^ The utility of these two scales can be further improved. For the ASCO VF to be widely adopted by patients, patient reported outcomes and patient preferences, which likely are different than those valued by clinicians, should be included.^33^ It may be useful to create a separate ASCO VF specifically for patients, which allow for various weighting options reflecting differences in preferences among patients. ASCO VF has a cumbersome process for scoring toxicity which will limit uptake. More recently, scores of many studies by ASCO VF NHB version 2 have been published, which can serve as a useful reference.^25^ Similarly, scores of ESMO MCBS version 1.1 are publicly available through the online score card on the ESMO website (https://www.esmo.org/score/cards). For ESMO MCBS to play a more prominent role in drug funding and health policy decisions, more comprehensive toxicity information need to be included, and formal cost analysis should be emphasized in addition to quantifying clinical benefit. Despite these limitations, both frameworks have continued to evolve with input from the stakeholder community. Further research is needed to assess the usage and uptake of the two scales by their intended audiences, and barriers preventing broader uptake. Finally, with updated analyses and newly available therapies, the true value of approved anticancer drugs may evolve over time.

There are several limitations of this study. We did not adjust for inflation for these drugs approvals across an 11‐year span. Other important aspects excluded from our cost estimation include drug wastage, biomarker testing, infusion times, requirement for clinical assessments and supportive care interventions to manage toxicity, indirect costs (such as costs related to productivity losses) and cost savings.

In conclusion, most FDA–approved anticancer drugs for advanced GI malignancies offer modest incremental clinical benefit yet have high prices, especially TT and IO agents. Delivery of high–quality cancer care within the context of cost–constrained health care systems will require patients, clinicians, payers, and policy makers to collectively address the value of approved anticancer drugs in this common disease site.

## CONFLICT OF INTEREST

None declared.
