# Supplementary figures and images for: Anticancer drugs approved by the Food and Drug Administration for gastrointestinal malignancies: Clinical benefit and price considerations
Source: Cancer Med. 2019 Mar 7;8(4):1584–93. doi: 10.1002/cam4.2058 (PMC6488126; doi:10.1002/cam4.2058)

A)

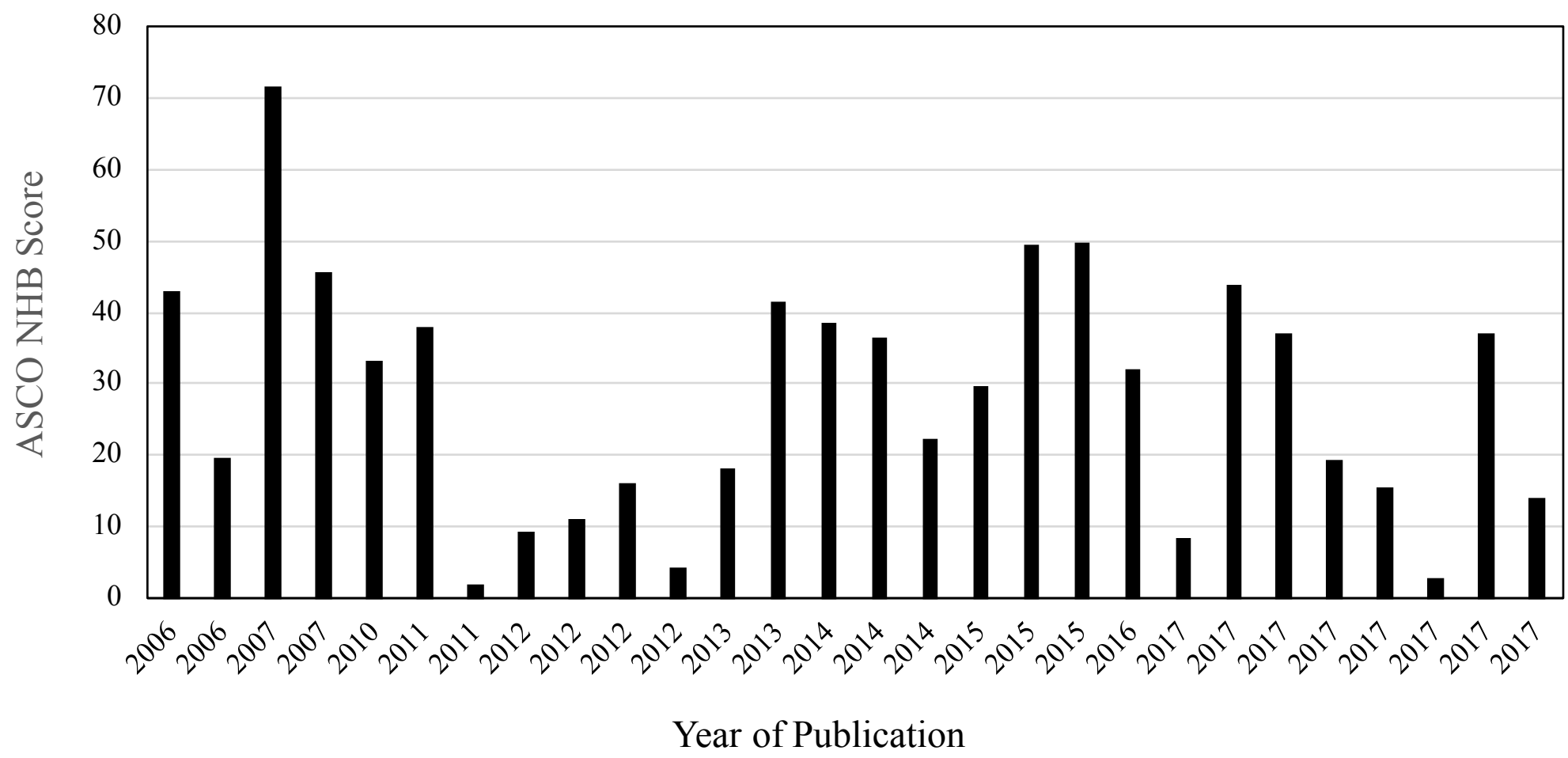

B)

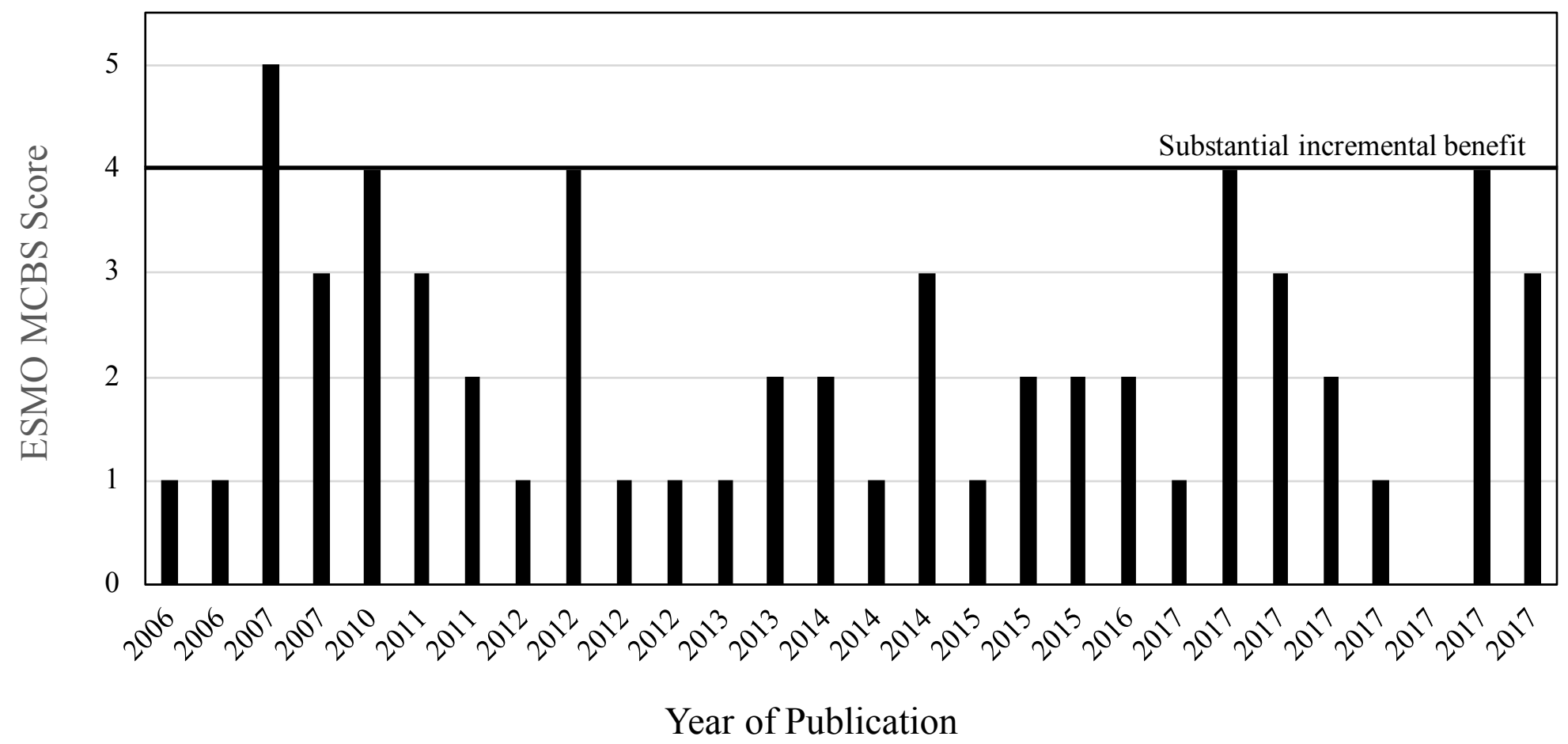

Supplement: Supplementary file 1 [file CAM4-8-1584-s001.pdf]
