# Supplementary material for: Anticancer drugs approved by the Food and Drug Administration for gastrointestinal malignancies: Clinical benefit and price considerations
Source: Cancer Med. 2019 Mar 7;8(4):1584–93. doi: 10.1002/cam4.2058 (PMC6488126; doi:10.1002/cam4.2058)
Supplement: Supplementary file 2 [file CAM4-8-1584-s002.docx]

Supplementary Table 1. Monthly average wholesale prices, median treatment durations, and estimated drug cost for four classes of newly FDA-approved GI cancer drugs.

| Classes of newly approved GI cancer drugs | Number of supporting trials | Median Monthly AWP (USD) | Median Treatment Duration (months) | Median Estimated Drug Costs per patient (USD) |
| --- | --- | --- | --- | --- |
| CT | 3 | $13,601 | 2.18 | $30,330 |
| Mab | 10 | $12,591 | 4.63 | $54,721 |
| IO | 7 | $14,659 | 6.60 | $98,208 |
| TT | 7 | $17,452 | 8.79 | $153,402 |

Abbreviations: CT, cytotoxic chemotherapy; Mab, monoclonal antibody; IO, immunotherapy; TT, oral targeted therapies; AWP, average wholesale price
