# Supplementary material for: Anticancer drugs approved by the Food and Drug Administration for gastrointestinal malignancies: Clinical benefit and price considerations
Source: Cancer Med. 2019 Mar 7;8(4):1584–93. doi: 10.1002/cam4.2058 (PMC6488126; doi:10.1002/cam4.2058)
Supplement: Supplementary file 3 [file CAM4-8-1584-s003.docx]

Discrepancies in ESMO MCBS v1.1 scores from other sources

| **Disease Setting** | **Trial, year** | **Treatment** | **ESMO MCBS score** | | | **Potential explanation** |
| --- | --- | --- | --- | --- | --- | --- |
|  |  |  | **Our score** | **Cherny 2018 JCO** [1] | **ESMO online scoring card** [2] |  |
| CRC | CO.17 2008 | Cetuximab vs BSC | 5 | 4 | 4 | Companion publication on QOL improvement [Au JCO 2009] – upgrade 1 level |
|  | Amado 2008 | Panitumumab vs BSC | 1 | 2 | - | Only PFS without OS/QOL benefit – downgrade 1 level |
|  | ECOG E3200 2007 | Bevacizumab +/- FOLFOX4 | 1 | 3 | 2 | OS HR0.75, 2.1mo - grade 1 on Form 2a OS<12m (HR>0.70 OR gain <1.5m) |
| pNET or IgNET | RADIANT4 2016 | Everolimus vs placebo | 2 | 3 | 3 | Only PFS without OS/QOL benefit – downgrade 1 level |

References

1. Cherny NI, Vries EGE de, Dafni U, Garrett-Mayer E, McKernin SE, Piccart M, Latino NJ, Douillard J-Y, Schnipper LE, Somerfield MR, Bogaerts J, Karlis D, Zygoura P, et al. Comparative Assessment of Clinical Benefit Using the ESMO-Magnitude of Clinical Benefit Scale Version 1.1 and the ASCO Value Framework Net Health Benefit Score. J Clin Oncol. 2018; : [Epub ahead of print].

2. Accessed from ESMO website <https://www.esmo.org/score/cards>
